# A Cross‐Sectional Study on Mental Health Burden Among Austrian Farmers: Sociodemographic, Work‐Related, and Health Behavior Factors

**DOI:** 10.1002/hsr2.71943

**Published:** 2026-02-26

**Authors:** Elke Humer, Christoph Pieh, Viktoria Neubauer

**Affiliations:** ^1^ Department for Psychosomatic Medicine and Psychotherapy University for Continuing Education Krems Krems Austria; ^2^ FFoQSI GmbH—Austrian Competence Centre for Feed and Food Quality, Safety and Innovation Tulln Austria; ^3^ Centre for Food Science and Veterinary Public Health, Clinical Department for Farm Animals and Food System Science University of Veterinary Medicine Vienna Vienna Austria

**Keywords:** farmers, health behaviors, mental health, occupational risk factors, sociodemographic risk factors

## Abstract

**Background and Aims:**

Although previous studies point to a high mental health burden in farmers, little is known about mental health in Austrian farmers, especially about factors associated with poor mental health.

**Methods:**

We assessed mental health in Austrian farmers and explored potential risk factors for poor mental health. A total of *N* = 2006 farmers (38.5% women; 61.4% men; a distribution closely mirroring the Austrian farming population) with a mean age of 45.04 ± 10.31 years took part in an online survey from October 2024 to February 2025 in which validated screening tools for symptoms of depression (PHQ‐9), anxiety (GAD‐7), sleep disorders (ISI‐2), perceived stress (PSS‐4), and alcohol abuse (CAGE) were applied. Gender, alongside other sociodemographic, work‐related, and behavioral variables, was included as a predictor in multivariable logistic regression analyses.

**Results:**

We found that higher adjusted odds for exceeding cut‐offs for clinically relevant mental health symptoms were associated with female gender, younger age, single individuals, physical inactivity outside the profession, smartphone usage, higher working hours, and financial insecurity.

**Conclusion:**

In conclusion, this study highlights the complex interplay between sociodemographic factors, work‐related variables, and health behaviors in determining the mental health outcomes of Austrian farmers. Targeted interventions that address risk factors, such as promoting physical activity and providing business/economic support, could help mitigate the mental health challenges faced by farmers.

## 1 Introduction

The mental health of farmers has gained increasing attention due to the unique and often severe challenges associated with agricultural work, which include financial instability, long working hours, and isolation [1, 2]. However, the existing body of research is characterized by significant heterogeneity and is predominantly based on studies from large‐scale, industrialized agricultural systems in countries such as the USA, Australia, and the UK [1]. The applicability of these findings to the distinct socio‐economic and cultural contexts of Central European countries like Austria remains largely unknown.

Austria's agricultural sector is defined by a number of unique characteristics that likely shape specific stress patterns and mental health risks. Structurally, it is dominated by small, family‐operated farms, often situated in alpine terrain that limits mechanization and increases physical labor, with the majority of farms run as part‐time operations requiring additional income work to ensure livelihood [3]. Systematically, Austrian farmers are integrated within the complex framework of the EU Common Agricultural Policy (CAP), facing pressures from market concentration and price volatility, while simultaneously holding a leading position in organic farming within the EU [4]. Culturally, deeply ingrained norms of self‐reliance and stoicism, alongside masculine ideals that can discourage help‐seeking, are believed to create significant barriers to addressing psychological distress in rural communities. These factors are compounded by a systemic lack of accessible, specialized mental health services in many rural areas [5, 6].

Recent data from companion studies indeed suggest that Austrian farmers experience elevated rates of depression, anxiety, insomnia, stress, and suicidal ideation compared to the general population [7, 8], yet empirical research into specific factors driving these disparities is lacking.

To comprehensively understand the mental health burden on Austrian farmers, a multi‐level perspective is essential. The socio‐ecological model provides a valuable framework for conceptualizing how factors at the individual, relationship, community, and structural levels interact to influence psychological well‐being. At the individual level, sociodemographic characteristics are crucial. Gender differences are well‐documented, with female farmers often reporting higher levels of anxiety, stress, and depression, potentially due to role strain and gendered coping mechanisms [6, 9, 10]. Age also plays a role; younger farmers may face stress from financial insecurity and intergenerational conflict on family farms [11], while older farmers may contend with issues related to physical health and succession [12]. Furthermore, relationships can serve as a key source of social support or buffering against occupational stress [13].

At the occupational and community level, work‐related factors are paramount. Financial instability is one of the most consistent predictors of poor mental health among farmers globally [2, 14]. The type of farming, such as conventional versus organic, may also be relevant, as different methods involve varying economic pressures, workloads, and philosophical approaches. Long working hours, a hallmark of the profession, can lead to chronic stress, sleep disturbances, and burnout [15]. Furthermore, full‐time farmers may be more vulnerable to work‐related stressors compared to part‐time farmers, who might have additional sources of income that provide financial stability [16].

Beyond work‐specific factors, health behaviors are critical. While farming is physically demanding, leisure‐time physical activity has been shown to offer distinct mental health benefits that occupational activity does not provide [17, 18]. Similarly, the role of technology, such as smartphone use, is ambivalent; it can foster social connection and provide access to resources, but excessive use may also be linked to poor mental health outcomes [19, 20].

Despite this understanding, a critical gap remains. No study has yet examined the complex interplay of these multi‐level factors—sociodemographic, occupational, and behavioral—within the unique Austrian context. This study, therefore, aims to provide the first comprehensive analysis of mental health and it correlates in a large, national sample of Austrian farmers. Guided by a socio‐ecological perspective, the specific objectives are to:

1. Assess the associations between sociodemographic variables (gender, age, education, partnership status, and region), and mental health indicators
2. Examine the link between work‐related factors (working hours, farm employment type, farming method, farm size, and financial situation), and mental health outcomes
3. Investigate the relationship between health behaviors (physical activity and smartphone use) and mental health indicators

By elucidating these associations, this research seeks to establish a crucial evidence base for developing targeted, context‐sensitive interventions and support policies to safeguard the well‐being of Austrian farmers.

## 2 Methods

### 2.1 Design

Between October 4, 2024, and February 28, 2025, an online survey targeting Austrian farmers was conducted via the LimeSurvey platform (LimeSurvey GmbH, Hamburg, Germany). Participation was entirely voluntary, with no financial or material incentives provided. Farmers were recruited through various channels, including announcements by agricultural chambers, farmer associations, and unions, as well as advertisements in agricultural magazines and newspapers. Moreover, the Austrian Ministry of Agriculture, dairy cooperatives, breeding associations, and other agricultural organizations helped promote the survey. A total of 2006 farmers fully completed the questionnaire. Out of the 2773 individuals who accessed the survey link, this resulted in a completion rate of 72.3%. The high participation rate was facilitated by the collaborative promotion of the survey through trusted national institutions and media, including the Agricultural Chambers, the Ministry of Agriculture, the Animal Health Service (Tiergesundheitsdienst), and stakeholder groups, and agricultural publications such as Top Agrar, which helped ensure broad reach and legitimacy.

Eligibility for inclusion in the final analysis required participants to be at least 18 years of age, currently residing in Austria, and self‐identifying as a farmer or being actively involved in managing an agricultural operation. Participants who did not complete the outcome measures were excluded from the analysis.

This study was conducted in alignment with the principles of the Declaration of Helsinki and was approved by the Ethics Commission of the Faculty of Psychotherapy Science and Psychology at Sigmund Freud University Vienna (Ethical approval number: XCXFA65WBWE@5490500, approval date: November 20, 2023). Participants gave electronic informed consent by completing the questionnaires to participate in the study.

### 2.2 Measures

#### 2.2.1 Sociodemographic Variables, Health Behaviors and Work‐Related Variables

Participants provided demographic information, including their gender (male, female, diverse), age (in years), highest level of education completed (ranging from no formal education to university degree), region of residence within Austria, and relationship status (single or in a relationship).

Health‐related behaviors were assessed via self‐report. Daily smartphone use (in hours) and levels of physical activity were documented. To evaluate physical activity, participants indicated on how many of the past 7 days they had been physically active for at least 60 min. To distinguish between activity during leisure time and physical exertion related to work, a separate question asked how many days they had engaged in at least 60 min of physical activity outside of their professional responsibilities. Smartphone usage was also self‐reported, with participants selecting from predefined categories: less than 1 h, 1–2 h, 3–4 h, 5–6 h, 7–8 h, or more than 8 h per day.

Additional questions for farmers included weekly working hours on the farm and in non‐agricultural jobs, type of employment in farming (full‐time or part‐time), farming method (organic, conventional, or integrated), whether livestock was kept, farm size (in hectares), and a subjective assessment of their financial situation (very good, good, modest, poor, or very poor).

#### 2.2.2 Depressive Symptoms (PHQ‐9)

Depressive symptoms were assessed using the depression module of the Patient Health Questionnaire (PHQ‐9) [21]. This self‐report instrument consists of nine items evaluating depressive symptoms experienced over the past 2 weeks. Participants rated each item on a four‐point scale, ranging from 0 (“not at all”) to 3 (“nearly every day”). The total score ranges from 0 to 27, with a threshold of 10 or higher indicating moderate, clinically relevant depressive symptoms [22]. In the present sample, the internal consistency of the PHQ‐9 was high, with a Cronbach's alpha of *α* = 0.87.

#### 2.2.3 Anxiety (GAD‐7)

Anxiety symptoms were measured using the Generalized Anxiety Disorder 7 (GAD‐7) scale, which consists of seven self‐report items [23]. The GAD‐7 assesses generalized anxiety symptoms experienced over the past 2 weeks, with responses given on a four‐point scale ranging from 0 (“not at all”) to 3 (“nearly every day”). The total score can range from 0 to 21, with a score of 10 or higher indicating clinically relevant anxiety symptoms [24]. The internal consistency of the GAD‐7 in the present sample was high, with a Cronbach's alpha of *α* = 0.91.

#### 2.2.4 Insomnia (ISI‐2)

Sleep quality was assessed using the two‐item version of the Insomnia Severity Index (ISI‐2) [25]. The self‐report items evaluate an individual's satisfaction with their current sleep patterns and the extent to which these patterns interfere with daily activities. Participants rated each item on a five‐point Likert scale, with responses ranging from 0 to 4. The total score on the ISI‐2 can range from 0 to 8, and a cut‐off score of 6 or higher has been proposed to indicate insomnia disorder [26]. In the present sample, the internal consistency of the ISI‐2 was *α* = 0.71.

#### 2.2.5 Perceived Stress (PSS‐4)

Perceived stress levels were measured using the four‐item Perceived Stress Scale (PSS‐4), a self‐report instrument. Participants rated their stress experiences on a five‐point Likert scale ranging from 0 (never) to 4 (very often) [27]. Items 2 and 3 were reverse‐coded. The total score on the PSS‐4 ranged from 0 to 16, with higher scores reflecting greater perceived stress. A score of 6 or higher was classified as indicating high stress levels [28]. In the present sample, the internal consistency yielded a reliability coefficient of *α* = 0.82.

#### 2.2.6 Alcohol Abuse (CAGE)

Alcohol abuse symptoms were assessed using the CAGE questionnaire [29], which consists of four yes/no questions addressing signs of alcoholism. These questions inquire about Cutting Down, Annoyance with Criticism, feelings of Guilt, and the need for an Eye‐Opener. The total score ranges from 0 to 4, with a score of 2 or higher suggesting alcohol abuse [30]. In the present sample, the internal consistency of the CAGE questionnaire was *α* = 0.60.

### 2.3 Statistical Analyses

The study design and reporting follow the Strengthening the Reporting of Observational Studies in Epidemiology (STROBE) guidelines [31].

Statistical analyses were performed with SPSS (version 26, IBM Corp in Armonk, NY, USA). All statistical analyses were conducted using two‐tailed tests, with a significance threshold set at *p* < 0.05.

All primary analyses were pre‐specified. The objective was to identify factors associated with clinically relevant mental health symptoms using multivariable binary logistic regression. No exploratory subgroup analyses were conducted.

To unravel factors linked to clinically relevant mental health symptoms, multivariable binary logistic regression was used. The dichotomized mental health variables (above vs. below cut‐off) for clinically relevant symptoms of depression, anxiety, insomnia, stress, and alcohol abuse served as the dependent variables in separate models.

The following pre‐defined predictors were included in all models: gender (female, male; participants identifying as diverse (*n* = 2) were excluded from these analyses due to an inadequate sample size for this category), age (in years), partnership status (single, in partnership), education (no school education or secondary school, apprenticeship, vocational secondary school, high school, university), region (West, East, South), physical activity (≥ 1 day/week, < 1 day/week physically active outside the of the job), smartphone usage (≥ 1 h/day, < 1 h/day), work spent farming (hours/week), work spent in other professions next to farming (hours/week), farm employment type (part‐time farming, full‐time farming), farming method (organic, conventional, integrated), farm size (< 10 ha, 10 to < 30 ha, 30 to < 50 ha, 50 to < 100 ha, ≥ 100 ha), animal husbandry (yes, no), and financial situation (very good, good, modest, poor, very poor).

Results are presented as adjusted odds ratios (aOR) with their associated 95% confidence intervals (CIs). The aOR represents the change in odds of the outcome for a one‐unit change in the predictor (for continuous variables) or for one category compared to the reference category (for categorical variables), while holding all other variables in the model constant. A 95% CI that does not include 1.0 indicates statistical significance at the *p* < 0.05 level.

## 3 Results

### 3.1 Study Sample Characteristics

A total of 2006 farmers took part in the study. An overview of their sociodemographic characteristics, health‐related behaviors, and occupational background is presented in Table 1. The average age of participants was 45.04 years (SD = 10.31), and 61.4% identified as male. Most respondents (73.8%) resided in western Austria, and 89.8% reported being in a relationship.

**Table 1:** Study sample characteristics (*N* = 2006).

	*N*	%
Gender		
Male	1232	61.4
Female	772	38.5
Diverse	2	0.1
Age, M (SD)	45.04 (10.31)	
Partnership status		
Single	204	10.2
In partnership	1802	89.8
Region		
Western Austria	1481	73.8
Eastern Austria	349	17.4
Southern Austria	176	8.8
Education		
No school education/secondary school	45	2.2
Apprenticeship	391	19.5
Vocational secondary school	910	45.4
High school	440	21.9
University	220	11.0
Farm employment type		
Full‐time farming	1321	65.9
Part‐time farming	685	34.1
Working hours		
Total hours per week in farming, M (SD)	49.94 (21.90)	
Total hours per week outside farming, M (SD)	12.73 (16.35)	
Farming method		
Organic	626	31.2
Conventional	419	20.9
Integrated	960	47.9
Others	1	0
Farm size		
Less than 10 ha	148	7.4
10 to less than 20 ha	311	15.5
20 to less than 30 ha	368	18.3
30 to less than 50 ha	582	29.0
50 to less than 100 ha	460	22.9
100 ha or more	137	6.8
Livestock		
No	149	7.4
Yes	1857	92.6
Physical activity outside farming		
0 days per week	736	36.7
≥ 1 day per week for ≥ 60 min	1270	63.3
Smartphone usage		
< 1 h/day	797	39.7
≥ 1 h/day	1209	60.3
Financial situation		
Very good	158	7.9
Good	644	32.1
Modest	736	36.7
Poor	341	17.0
Very poor	127	6.3

*Note:* The regions were classified according to NUTS 1 (Nomenclature of territorial units for statistics) into three major socio‐economic regions (Eastern Austria: Burgenland, Lower Austria, Vienna; Southern Austria: Carinthia, Styria; Western Austria: Upper Austria, Salzburg, Tyrol, Vorarlberg). Vocational secondary school typically includes specialized technical or agricultural training.

Almost two‐thirds (65.9%) of participants were full‐time farmers. The participants worked on average 49.94 ± 21.90 h per week on the farm and 12.37 ± 16.35 in other professions. Almost one third (31.2%) were organic farmers. Only a minority of 7.4% were not practicing animal husbandry. Most farmers estimated their financial situation as good (32.1%) or modest (36.7%).

Regarding health behaviors, 63.3% of farmers were physically active outside of the profession for at least 1 h per day on at least 1 day per week, and the majority (60.3%) spent more than 1 h per day on the smartphone.

### 3.2 Association of Sociodemographic Factors, Health Behaviors, and Work‐Related Variables With Mental Health Indicators in Farmers

Detailed statistical results are summarized in Supporting Information S1: Tables 1–5. Only significant findings are reported in the following.

### 3.3 Sociodemographic Variables

Being female was linked to a greater likelihood of experiencing clinically relevant symptoms of depression (aOR = 1.29; 95% CI: 1.01–1.64), anxiety (aOR = 1.45; 95% CI: 1.15–1.83), and elevated stress levels (aOR = 1.31; 95% CI: 1.03–1.67). In contrast, women showed reduced odds of exhibiting symptoms indicative of alcohol abuse (aOR = 0.32; 95% CI: 0.23–0.44).

With increasing age, the probability of reporting high stress symptoms decreased slightly (aOR = 0.99; 95% CI: 0.977–0.999), as did the likelihood of alcohol‐related problems (aOR = 0.98; 95% CI: 0.97–0.99).

Being in a partnership was associated with lower odds for depressive symptoms (aOR: 0.56; 95% CI: 0.40; 0.79) and anxiety (aOR: 0.70; 95% CI: 0.50; 0.98).

Farmers from Eastern Austria had higher odds for depression (aOR: 1.56; 95% CI: 1.16; 2.10) and high stress (aOR: 1.48; 95% CI: 1.08; 2.04) compared to those from Western Austria.

### 3.4 Health Behaviors

**Figure 1:**
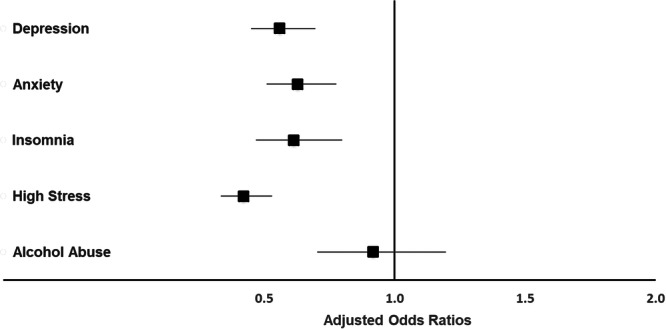
Adjusted odds ratios for clinically relevant symptoms of depression, anxiety, insomnia, stress, and alcohol abuse in physically active (≥ 1 day/week physically active outside of the job; *n* = 1269) versus inactive (< 1 day/week physically active outside of the job; *n* = 735) farmers. The multivariable model was adjusted for age, gender, education, region, partnership status, smartphone usage, workload, farm employment status, farm type, animal husbandry, farm size, and estimated financial situation. An adjusted odds ratio of 1 indicates no difference. Adjusted odds ratios < 1 indicate a lower relative risk in physically active farmers compared to physically inactive farmers. Confidence intervals (horizontal lines) crossing 1 (vertical line) indicate no significant difference between physically active and inactive farmers.

Engaging in physical activity—defined as being active for at least 60 min per day on one or more days per week outside of farm work—was linked to a lower likelihood of scoring above the clinical cut‐offs for symptoms of depression (aOR = 0.56; 95% CI: 0.45–0.70), anxiety (aOR = 0.63; 95% CI: 0.51–0.78), insomnia (aOR = 0.61; 95% CI: 0.47–0.80), and stress (aOR = 0.42; 95% CI: 0.33–0.53), as shown in Figure 1.

In terms of smartphone use, individuals who reported using their phones for at least 1 h per day had reduced odds of experiencing clinically relevant anxiety symptoms (aOR = 0.77; 95% CI: 0.62–0.96) but showed an increased likelihood of alcohol misuse (aOR = 1.34; 95% CI: 1.01–1.78).

### 3.5 Work‐Related Variables

With regard to occupational variables, a higher overall workload was linked to greater odds of reporting clinically relevant symptoms of depression (aOR = 1.01; 95% CI: 1.01–1.02), anxiety (aOR = 1.02; 95% CI: 1.01–1.03), insomnia (aOR = 1.01; 95% CI: 1.004–1.02), and elevated stress (aOR = 1.01; 95% CI: 1.01–1.02). Additionally, more working hours in non‐farming occupations were associated with increased risks for symptoms of depression (aOR = 1.01; 95% CI: 1.003–1.02), anxiety (aOR = 1.01; 95% CI: 1.004–1.02), and insomnia (aOR = 1.01; 95% CI: 1.003–1.03).

Participants engaged in animal husbandry showed reduced odds of clinically relevant anxiety symptoms (aOR = 0.65; 95% CI: 0.42–0.99). Compared to organic farming, working in conventional (aOR = 1.64; 95% CI: 1.22–2.21) or integrated (aOR = 1.34; 95% CI: 1.06–1.70) agricultural systems was associated with an increased risk of high stress.

**Figure 2:**
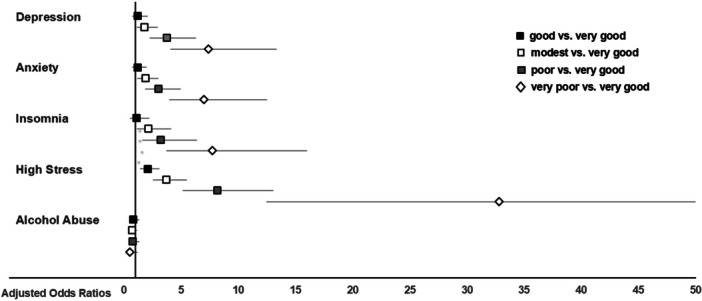
Adjusted odds ratios for clinically relevant symptoms of depression, anxiety, insomnia, stress, and alcohol abuse in farmers estimating their financial situation as either good (*n* = 643), modest (*n* = 736), poor (*n* = 341), or very poor (*n* = 126) compared to very good (*n* = 158). The multivariable model was adjusted for age, gender, education, region, partnership status, smartphone usage, workload, farm employment status, farm type, animal husbandry, farm size, estimated financial situation. An adjusted odds ratio of 1 indicates no difference. Adjusted odds ratios > 1 indicate a higher relative risk in farmers estimating their financial situation as either good, modest, poor, or very poor compared to those estimating their financial situation as very good. Confidence intervals (horizontal lines) crossing 1 (vertical line) indicate no significant difference between different estimations of their financial situation.

Furthermore, financial strain was consistently linked to higher probabilities of exceeding the clinical thresholds for depression, anxiety, insomnia, and stress symptoms, as illustrated in Figure 2.

In contrast, neither the type of employment on the farm (full‐time vs. part‐time) nor farm size was significantly associated with any of the examined mental health outcomes.

## 4 Discussion

This study provides the first national assessment of factors associated with poor mental health among Austrian farmers using validated psychometric tools. We interpret our findings through a socio‐ecological and stress process lens [32, 33]. This framework posits that mental health outcomes result from the interplay between external stressors (e.g., financial strain, climate events) and internal resources (e.g., coping strategies, social support) which are shaped by sociodemographic position. Our results elucidate how risk and protective factors at individual, occupational, and community levels converge to influence the mental health of this vital occupational group.

### 4.1 Sociodemographic Factors: A Nuanced Gendered Landscape

Our analysis confirms gender as a significant predictor, with female farmers reporting higher odds of depression, anxiety, and stress. This aligns with general population trends and studies in agriculture, often attributed to women's dual burden of farm and domestic labor and gendered role strain [9, 34, 35, 36]. These findings are further contextualized by the Role Congruity Theory; qualitative research indicates that women in agriculture experience stress from gender stereotyping, misogyny, and a perceived incongruity with the male‐dominated sector, leading to behavioral adaptations like overcompensation, which may exacerbate stress [37]. It also resonates with a recent call to prioritize research on women in agriculture in the Global North [38]. However, a complete understanding requires situating this finding within the broader, gendered context of mental health. While women generally report higher rates of internalizing disorders (depression, anxiety), men—particularly in rural, masculine‐coded professions like farming—exhibit higher rates of suicide mortality and often externalize stress through risk‐taking and substance abuse [39]. This “gender‐paradox” in suicidality suggests that our finding of higher reported symptoms among women may not fully capture the severity of distress, which can be masked by help‐seeking reluctance and norms of stoicism [40, 41]. Research specifically in agricultural communities shows that higher perceived stress is a direct predictor of binge drinking behavior, highlighting a critical pathway for male externalization of distress [42]. Therefore, the lower odds ratios for men in our models may reflect under‐reporting or different symptom manifestation (e.g., higher alcohol abuse, as seen in our study) rather than lower risk. This underscores the need for gender‐sensitive approaches: targeted support for women's documented stressors and destigmatized, male‐appropriate mental health services that address masculine socialization and isolation [43].

Other sociodemographic factors further delineate vulnerability. The protective effect of partnership status against depression and anxiety robustly supports the stress‐buffering hypothesis, where intimate social support mitigates psychological strains [44, 45]. This highlights the mental health value of strong social connections in a potentially isolating profession. Conversely, the vulnerability of younger farmers to alcohol abuse challenges some previous findings [46], but aligns with research linking early‐career, financial pressure, and maladaptive coping [2]. This suggests a shift in risk patterns, where contemporary economic pressures disproportionately impact newer entrants.

Furthermore, the significant regional disparity, with higher odds of depression and stress in Eastern Austria, can be directly contextualized with the stress process model as an acute, macro‐level stressor. The severe flooding and storms in autumn 2024 [47, 48] acted as a profound environmental and economic shock, demonstrating how climate‐related disruptions directly translate into population‐level mental health crises.

### 4.2 Health Behaviors and Work‐Related Factors: Modifiable Pathways

Our findings identify modifiable pathways for intervention. The consistent protective effect of leisure‐time physical activity against depression, anxiety, insomnia, and stress underscores that physically demanding farm work does not confer the same psychological benefits as voluntary exercise, which likely offers mastery experience and stress detachment [18, 20]. This distinction is crucial for health promotion.

The ambivalent role of smartphone use (> 1 h/day linked to lower anxiety, but higher alcohol abuse) reflects the dual function as a tool for connectivity and a medium for maladaptive escape [49, 50, 51]. This nuance is vital for designing digital well‐being guidelines for farming communities.

Core occupational stressors powerfully predict outcomes. Long working hours and multiple job holdings directly represent chronic role strain, depleting energy reserves, and limiting recovery consistent with job‐demand models [2]. Most critically, financial insecurity was the strongest correlate across almost all mental health measures, confirming its role as a foundational stressor within the farming stress process.

The mechanisms linking financial strain to poor mental health are multifaceted. Pervasive uncertainty and threat to livelihood [2, 52] are fueled by volatile input costs, fluctuating market prices, and often significant debt burdens [53]. Additionally, pressures related to farm succession can compound this strain, intertwining economic anxiety with familial and identity concerns [54]. This stressor is exacerbated by its policy and historical context. Farmers operate within the complex framework of the CAP, where reforms and shifts in subsidy structures can create profound insecurity [55]. Concurrently, global market shocks and the rising frequency of climate‐related disasters—such as the 2024 floods in Eastern Austria [47, 48]— directly undermine economic stability.

Critically, financial strain likely does not operate in isolation. It may act as a key mediator, helping to explain the regional disparities observed in this study. The acute climate shocks in Eastern Austria, for instance, presumably translated into substantial economic losses, thereby exacerbating mental health symptoms through a financial pathway. Furthermore, financial strain can moderate the impact of other stressors, where pre‐existing economic precarity magnifies the psychological toll of workload or family conflict. Therefore, addressing financial insecurity is not merely one intervention among many, but potentially the most powerful lever for mitigating the mental health burden on Austrian farmers.

### 4.3 Theoretical Integration and Practical Implications

Theoretically, our results affirm the value of the stress process model. Factors like financial strain and climate disasters operate as primary stressors. Gender, age, and partnership status shape differential exposure and vulnerability to these stressors, as well as access to resources (e.g., coping through exercise, partner support). The outcomes—from internalizing disorders to alcohol abuse—represent the manifestations of this process.

These insights lead to concrete, multi‐level implications. In terms of public health and clinical practice, this means developing targeted outreach programs that promote structured physical activity schemes tailored to farmers, creating financial counseling and stress‐management workshops, and train rural healthcare providers in gender‐specific (particularly male‐engaging) mental health identification and referral strategies. On the agricultural and policy front, it is necessary to advocate for policies that address root economic stressors. This includes stabilizing farm incomes through fair pricing mechanisms and resilient subsidy frameworks, supporting farm succession planning to alleviate intergenerational stress, and integrating mental health risk assessments into existing agricultural extension services. At the community level, fostering peer‐support networks can help reduce isolation, especially for male, single, and young farmers.

Future longitudinal research should track how these mental health patterns evolve with changing policies and climate, and qualitative studies are needed to deeply explore the lived experience and coping mechanisms of both male and female farmers.

### 4.4 Limitations

While this study provides important insights into the mental health of Austrian farmers, there are several limitations to consider. First, the cross‐sectional design of the study limits the ability to draw causal conclusions. Longitudinal studies are needed to better understand the temporal relationships between sociodemographic, work‐related factors, health behaviors, and mental health outcomes. Additionally, the reliance on self‐reported data may have introduced response biases, particularly in the reporting of sensitive topics like alcohol use. Future studies could incorporate objective measures of alcohol consumption and mental health symptoms to corroborate self‐reported data. Another limitation is the focus on farmers who are already engaged in agriculture, potentially overlooking those who may have exited the profession due to mental health issues or other reasons. This could result in an underestimation of the mental health challenges faced by farmers, as those who leave the profession may be experiencing even greater distress. Finally, while the study sample was large and diverse in terms of age and region, it may not fully capture the experiences of farmers in more remote or disadvantaged areas. Further research should seek to include these groups to ensure that the findings are representative of the entire farming population.

## 5 Conclusion

As the first national study to assess Austrian farmers' mental health using validated psychometric tools, this research advances a socio‐ecological understanding of how mental health is shaped within agricultural contexts. By applying a stress‐process lens, we move beyond descriptive risk factors to illustrate how macro‐level stressors (financial insecurity, climate‐related disruptions) interact with social positions (gender, age, partnership) and individual behaviors (physical activity, smartphone use) to produce distinct mental health outcomes.

These findings directly inform both future research and policy. For researchers, they establish a crucial national baseline and call for longitudinal studies to track evolving stressors, as well as qualitative work to explore the gendered and contextual nuances of coping and help‐seeking.

For policymakers and practitioners, the evidence underscores the necessity of integrated, cross‐sectoral strategies. Actionable steps should include agricultural and economic policy to stabilize farm incomes and reduce financial precarity as a foundational intervention, as well as public health outreach that is gender‐sensitive, that promotes physical activity programs, and that supports healthy technology use through practical digital‐literacy guidance. Finally, integrated support structures are needed, in which agricultural extension services, healthcare providers, and rural community organizations collaborate to identify at‐risk individuals and connect them to appropriate resources.

Supporting farmers' mental health is not merely a clinical or social concern—it is central to the resilience and sustainability of Austria's food systems. This study provides the empirical foundation for turning that recognition into coordinated action across health, agricultural, and social policy sectors.

## Author Contributions

**Elke Humer:** conceptualization, investigation, writing – original draft, methodology, visualization, software, formal analysis, project administration, data curation, supervision, resources. **Christoph Pieh:** writing – review and editing. **Viktoria Neubauer:** conceptualization, investigation, methodology, validation, writing – review and editing.

## Funding

The authors received no specific funding for this work.

## Conflicts of Interest

The authors declare no conflicts of interest.

## Transparency Statement

The corresponding author, Elke Humer, affirms that this manuscript is an honest, accurate, and transparent account of the study being reported; that no important aspects of the study have been omitted; and that any discrepancies from the study as planned (and, if relevant, registered) have been explained.

## Supporting information


**Table S1.** Adjusted odds ratios for depressive symptoms (assessed with the Patient Health Questionnaire‐9 (PHQ‐9)) in farmers (n = 2.004). **Table S2.** Adjusted odds ratios for anxiety symptoms (assessed with the Generalized Anxiety Scale‐7 (GAD‐7)) in farmers (n = 2.004). **Table S3.** Adjusted odds ratios for insomnia symptoms (assessed with the Insomnia Severity Scale‐2 (ISS‐2)) in farmers (n = 2.004). **Table S4.** Adjusted odds ratios for stress symptoms (assessed with the Perceived Stress Scale‐4 (PSS‐4)) in farmers (n = 2.004). **Table S5.** Adjusted odds ratios for symptoms of alcohol abuse (assessed with the CAGE questionnaire) in farmers (n = 2.004).

## Data Availability

The data sets used and analyzed during the current study are available from the corresponding author on reasonable request.
