# Supplementary material for: A Cross‐Sectional Study on Mental Health Burden Among Austrian Farmers: Sociodemographic, Work‐Related, and Health Behavior Factors
Source: Health Sci Rep. 2026 Feb 26;9(3):e71943. doi: 10.1002/hsr2.71943 (PMC12946656; doi:10.1002/hsr2.71943)
Supplement: Supplementary file 1 — Table S1. Adjusted odds ratios for depressive symptoms (assessed with the Patient Health Questionnaire‐9 (PHQ‐9)) in farmers (n = 2.004). Table S2. Adjusted odds ratios for anxiety symptoms (assessed with the Generalized Anxiety Scale‐7 (GAD‐7)) in farmers (n = 2.004). Table S3. Adjusted odds ratios for insomnia symptoms (assessed with the Insomnia Severity Scale‐2 (ISS‐2)) in farmers (n = 2.004). Table S4. Adjusted odds ratios for stress symptoms (assessed with the Perceived Stress Scale‐4 (PSS‐4)) in farmers (n = 2.004). Table S5. Adjusted odds ratios for symptoms of alcohol abuse (assessed with the CAGE questionnaire) in farmers (n = 2.004). [file HSR2-9-e71943-s001.docx]

**Suppl. Table 1.** Adjusted odds ratios for depressive symptoms (assessed with the Patient Health Questionnaire-9 (PHQ-9)) in farmers (n = 2.004).

| Predictor | | aOR | 95% Confidence Interval | | p-value |
| --- | --- | --- | --- | --- | --- |
|  | Gender (male vs. female) | 1.286 | 1.010 | 1.637 | .041 |
|  | Age | 1.005 | .993 | 1.016 | .42 |
|  | Partnership (in partnership vs. single) | .559 | .397 | .785 | <.001 |
|  | Education |  |  |  | .38 |
|  | Education (Apprenticeship vs. no/secondary school) | .649 | .325 | 1.295 | .22 |
|  | Education (Vocational secondary school vs. no/secondary school) | .670 | .342 | 1.312 | .24 |
|  | Education (High school vs. no/secondary school) | .533 | .265 | 1.071 | .077 |
|  | Education (University vs. no/secondary school) | .628 | .303 | 1.301 | .21 |
|  | Region |  |  |  | .011 |
|  | Region (East vs. West) | 1.559 | 1.156 | 2.104 | .004 |
|  | Region (South vs. West) | 1.276 | .879 | 1.853 | .20 |
|  | Physical active outside farming ≥ 1d/week for ≥ 60 min | .559 | .449 | .696 | <.001 |
|  | Smartphone usage ≥ 1 h/d | .938 | .748 | 1.178 | .58 |
|  | Work spent farming | 1.014 | 1.008 | 1.021 | <.001 |
|  | Work spent outside farming | 1.012 | 1.003 | 1.022 | .009 |
|  | Farm employment type (part-time vs. full time) | 1.145 | .835 | 1.571 | .40 |
|  | Farming method |  |  |  | .86 |
|  | Farming method (conventional vs. organic) | 1.074 | .797 | 1.447 | .64 |
|  | Farming method (integrated vs. organic) | 1.061 | .830 | 1.356 | .64 |
|  | Farm size |  |  |  | .21 |
|  | Farm size (10 to < 20 ha vs. <10 ha) | 1.079 | .665 | 1.749 | .76 |
|  | Farm size (20 to < 30 ha vs. <10 ha) | .792 | .486 | 1.290 | .35 |
|  | Farm size (30 to < 50 ha vs. <10 ha) | 1.215 | .760 | 1.943 | .42 |
|  | Farm size (50 to < 100 ha vs. <10 ha) | 1.180 | .720 | 1.935 | .51 |
|  | Farm size (≥100 ha vs. <10 ha) | 1.060 | .577 | 1.947 | .85 |
|  | Animal husbandry (yes vs. no) | .956 | .612 | 1.493 | .84 |
|  | Financial situation |  |  |  | <.001 |
|  | Financial situation (good vs. very good) | 1.297 | .793 | 2.123 | .30 |
|  | Financial situation (modest vs. very good) | 1.885 | 1.166 | 3.047 | .010 |
|  | Financial situation (poor vs. very good) | 4.119 | 2.496 | 6.797 | <.001 |
|  | Financial situation (very poor vs. very good) | 7.641 | 4.247 | 13.750 | <.001 |

Note: The regions were classified according to NUTS 1 (Nomenclature of territorial units for statistics) into three major socio-economic regions (Eastern Austria: Burgenland. Lower Austria. Vienna; Southern Austria: Carinthia. Styria; Western Austria: Upper Austria. Salzburg. Tyrol. Vorarlberg).

The binomial logistic regression model was statistically significant. χ²(26) = 245.926. p < .001. Overall percentage of accuracy in classification was 74.7% and the amount of explained variance. as shown by Nagelkerke’s R². was .166.

**Suppl. Table 2.** Adjusted odds ratios for anxiety symptoms (assessed with the Generalized Anxiety Scale-7 (GAD-7)) in farmers (n = 2.004).

| Predictor | | aOR | 95% Confidence Interval | | p-value |
| --- | --- | --- | --- | --- | --- |
|  | Gender (male vs. female) | 1.449 | 1.148 | 1.830 | .002 |
|  | Age | .993 | .982 | 1.004 | .20 |
|  | Partnership (in partnership vs. single) | .699 | .499 | .979 | .037 |
|  | Education |  |  |  | .98 |
|  | Education (Apprenticeship vs. no/secondary school) | 1.074 | .524 | 2.203 | .85 |
|  | Education (Vocational secondary school vs. no/secondary school) | 1.069 | .530 | 2.155 | .85 |
|  | Education (High school vs. no/secondary school) | .996 | .484 | 2.051 | .99 |
|  | Education (University vs. no/secondary school) | 1.112 | .525 | 2.354 | .78 |
|  | Region |  |  |  | .11 |
|  | Region (East vs. West) | 1.216 | .904 | 1.636 | .20 |
|  | Region (South vs. West) | 1.409 | .987 | 2.012 | .059 |
|  | Physical active outside farming ≥ 1d/week for ≥ 60 min | .629 | .509 | .777 | <.001 |
|  | Smartphone usage ≥ 1 h/d | .771 | .619 | .960 | .020 |
|  | Work spent farming | 1.020 | 1.013 | 1.026 | <.001 |
|  | Work spent outside farming | 1.013 | 1.004 | 1.022 | .004 |
|  | Farm employment type (part-time vs. full time) | 1.241 | .915 | 1.682 | .16 |
|  | Farming method |  |  |  | .94 |
|  | Farming method (conventional vs. organic) | .956 | .716 | 1.275 | .76 |
|  | Farming method (integrated vs. organic) | .967 | .764 | 1.223 | .78 |
|  | Farm size |  |  |  | .91 |
|  | Farm size (10 to < 20 ha vs. <10 ha) | 1.046 | .655 | 1.670 | .85 |
|  | Farm size (20 to < 30 ha vs. <10 ha) | 1.016 | .638 | 1.618 | .95 |
|  | Farm size (30 to < 50 ha vs. <10 ha) | 1.205 | .765 | 1.896 | .42 |
|  | Farm size (50 to < 100 ha vs. <10 ha) | 1.152 | .714 | 1.857 | .56 |
|  | Farm size (≥100 ha vs. <10 ha) | 1.137 | .633 | 2.041 | .67 |
|  | Animal husbandry (yes vs. no) | .646 | .423 | .988 | .044 |
|  | Financial situation |  |  |  | <.001 |
|  | Financial situation (good vs. very good) | 1.269 | .799 | 2.014 | .31 |
|  | Financial situation (modest vs. very good) | 1.953 | 1.245 | 3.064 | .004 |
|  | Financial situation (poor vs. very good) | 3.252 | 2.022 | 5.230 | <.001 |
|  | Financial situation (very poor vs. very good) | 7.538 | 4.256 | 13.351 | <.001 |

Note: The regions were classified according to NUTS 1 (Nomenclature of territorial units for statistics) into three major socio-economic regions (Eastern Austria: Burgenland. Lower Austria. Vienna; Southern Austria: Carinthia. Styria; Western Austria: Upper Austria. Salzburg. Tyrol. Vorarlberg).

The binomial logistic regression model was statistically significant. χ²(26) = 230.893. p < .001. Overall percentage of accuracy in classification was 73.0% and the amount of explained variance. as shown by Nagelkerke’s R². was .153.

**Suppl. Table 3.** Adjusted odds ratios for insomnia symptoms (assessed with the Insomnia Severity Scale-2 (ISS-2)) in farmers (n = 2.004).

| Predictor | | aOR | 95% Confidence Interval | | p-value |
| --- | --- | --- | --- | --- | --- |
|  | Gender (male vs. female) | 1.285 | .959 | 1.720 | .093 |
|  | Age | 1.009 | .996 | 1.023 | .18 |
|  | Partnership (in partnership vs. single) | .810 | .531 | 1.235 | .33 |
|  | Education |  |  |  | .36 |
|  | Education (Apprenticeship vs. no/secondary school) | .667 | .299 | 1.489 | .32 |
|  | Education (Vocational secondary school vs. no/secondary school) | .686 | .315 | 1.494 | .34 |
|  | Education (High school vs. no/secondary school) | .586 | .259 | 1.325 | .20 |
|  | Education (University vs. no/secondary school) | .922 | .399 | 2.130 | .85 |
|  | Region |  |  |  | .33 |
|  | Region (East vs. West) | .944 | .649 | 1.374 | .76 |
|  | Region (South vs. West) | 1.354 | .877 | 2.091 | .17 |
|  | Physical active outside farming ≥ 1d/week for ≥ 60 min | .613 | .469 | .799 | <.001 |
|  | Smartphone usage ≥ 1 h/d | 1.081 | .820 | 1.426 | .58 |
|  | Work spent farming | 1.012 | 1.004 | 1.020 | .002 |
|  | Work spent outside farming | 1.014 | 1.003 | 1.025 | .011 |
|  | Farm employment type (part-time vs. full time) | .782 | .532 | 1.149 | .21 |
|  | Farming method |  |  |  | .94 |
|  | Farming method (conventional vs. organic) | .985 | .687 | 1.413 | .94 |
|  | Farming method (integrated vs. organic) | .951 | .707 | 1.279 | .74 |
|  | Farm size |  |  |  | .66 |
|  | Farm size (10 to < 20 ha vs. <10 ha) | 1.456 | .791 | 2.678 | .23 |
|  | Farm size (20 to < 30 ha vs. <10 ha) | 1.044 | .563 | 1.934 | .89 |
|  | Farm size (30 to < 50 ha vs. <10 ha) | 1.196 | .656 | 2.181 | .56 |
|  | Farm size (50 to < 100 ha vs. <10 ha) | 1.154 | .617 | 2.160 | .65 |
|  | Farm size (≥100 ha vs. <10 ha) | 1.417 | .677 | 2.966 | .36 |
|  | Animal husbandry (yes vs. no) | .769 | .451 | 1.310 | .33 |
|  | Financial situation |  |  |  | <.001 |
|  | Financial situation (good vs. very good) | 1.028 | .531 | 1.991 | .94 |
|  | Financial situation (modest vs. very good) | 2.004 | 1.066 | 3.767 | .031 |
|  | Financial situation (poor vs. very good) | 3.062 | 1.596 | 5.876 | <.001 |
|  | Financial situation (very poor vs. very good) | 7.182 | 3.555 | 14.509 | <.001 |

Note: The regions were classified according to NUTS 1 (Nomenclature of territorial units for statistics) into three major socio-economic regions (Eastern Austria: Burgenland. Lower Austria. Vienna; Southern Austria: Carinthia. Styria; Western Austria: Upper Austria. Salzburg. Tyrol. Vorarlberg).

The binomial logistic regression model was statistically significant. χ²(26) = 145.954. p < .001. Overall percentage of accuracy in classification was 84.9% and the amount of explained variance. as shown by Nagelkerke’s R². was .122.

| **Suppl. Table 4.** Adjusted odds ratios for stress symptoms (assessed with the Perceived Stress Scale-4 (PSS-4)) in farmers (n = 2.004).   \| Predictor \| \| aOR \| 95% Confidence Interval \| \| p-value \| \| --- \| --- \| --- \| --- \| --- \| --- \| \|  \| Gender (male vs. female) \| 1.314 \| 1.034 \| 1.668 \| .025 \| \| Age \| .988 \| .977 \| .999 \| .033 \| \| Partnership (in partnership vs. single) \| .943 \| .663 \| 1.343 \| .75 \| \| Education \|  \|  \|  \| .103 \| \| Education (Apprenticeship vs. no/secondary school) \| .627 \| .289 \| 1.361 \| .24 \| \| Education (Vocational secondary school vs. no/secondary school) \| .619 \| .290 \| 1.321 \| .22 \| \| Education (High school vs. no/secondary school) \| .477 \| .220 \| 1.035 \| .061 \| \| Education (University vs. no/secondary school) \| .462 \| .208 \| 1.027 \| .058 \| \| Region \|  \|  \|  \| .037 \| \| Region (East vs. West) \| 1.481 \| 1.077 \| 2.035 \| .016 \| \| Region (South vs. West) \| 1.268 \| .874 \| 1.838 \| .21 \| \| Physical active outside farming ≥ 1d/week for ≥ 60 min \| .421 \| .334 \| .530 \| <.001 \| \| Smartphone usage ≥ 1 h/d \| 1.067 \| .852 \| 1.336 \| .57 \| \| Work spent farming \| 1.013 \| 1.006 \| 1.020 \| <.001 \| \| Work spent outside farming \| 1.009 \| 1.000 \| 1.019 \| .054 \| \| Farm employment type (part-time vs. full time) \| 1.154 \| .846 \| 1.574 \| .37 \| \| Farming method \|  \|  \|  \| .003 \| \| Farming method (conventional vs. organic) \| 1.641 \| 1.220 \| 2.208 \| .001 \| \| Farming method (integrated vs. organic) \| 1.344 \| 1.062 \| 1.700 \| .014 \| \| Farm size \|  \|  \|  \| .40 \| \| Farm size (10 to < 20 ha vs. <10 ha) \| 1.180 \| .754 \| 1.846 \| .47 \| \| Farm size (20 to < 30 ha vs. <10 ha) \| 1.332 \| .851 \| 2.086 \| .21 \| \| Farm size (30 to < 50 ha vs. <10 ha) \| 1.496 \| .961 \| 2.329 \| .074 \| \| Farm size (50 to < 100 ha vs. <10 ha) \| 1.223 \| .767 \| 1.948 \| .40 \| \| Farm size (≥100 ha vs. <10 ha) \| 1.555 \| .859 \| 2.814 \| .15 \| \| Animal husbandry (yes vs. no) \| .700 \| .448 \| 1.094 \| .12 \| \| Financial situation \|  \|  \|  \| <.001 \| \| Financial situation (good vs. very good) \| 2.047 \| 1.404 \| 2.984 \| <.001 \| \| Financial situation (modest vs. very good) \| 3.678 \| 2.519 \| 5.369 \| <.001 \| \| Financial situation (poor vs. very good) \| 8.452 \| 5.356 \| 13.337 \| <.001 \| \| Financial situation (very poor vs. very good) \| 32.616 \| 12.415 \| 85.690 \| <.001 \|   Note: The regions were classified according to NUTS 1 (Nomenclature of territorial units for statistics) into three major socio-economic regions (Eastern Austria: Burgenland. Lower Austria. Vienna; Southern Austria: Carinthia. Styria; Western Austria: Upper Austria. Salzburg. Tyrol. Vorarlberg).  The binomial logistic regression model was statistically significant. χ²(26) = 345.127. p < .001. Overall percentage of accuracy in classification was 72.7% and the amount of explained variance. as shown by Nagelkerke’s R². was .220.  **Suppl. Table 5.** Adjusted odds ratios for symptoms of alcohol abuse (assessed with the CAGE questionnaire) in farmers (n = 2.004).   \| Predictor \| \| aOR \| 95% Confidence Interval \| \| p-value \| \| --- \| --- \| --- \| --- \| --- \| --- \| \|  \| Gender (male vs. female) \| .322 \| .233 \| .444 \| <.001 \| \| Age \| .980 \| .967 \| .993 \| .002 \| \| Partnership (in partnership vs. single) \| 1.026 \| .698 \| 1.508 \| .90 \| \| Education \|  \|  \|  \| .062 \| \| Education (Apprenticeship vs. no/secondary school) \| .501 \| .219 \| 1.143 \| .10 \| \| Education (Vocational secondary school vs. no/secondary school) \| .414 \| .186 \| .920 \| .030 \| \| Education (High school vs. no/secondary school) \| .600 \| .264 \| 1.362 \| .22 \| \| Education (University vs. no/secondary school) \| .594 \| .253 \| 1.397 \| .23 \| \| Region \|  \|  \|  \| .29 \| \| Region (East vs. West) \| .982 \| .677 \| 1.424 \| .92 \| \| Region (South vs. West) \| 1.380 \| .910 \| 2.093 \| .13 \| \| Physical active outside farming ≥ 1d/week for ≥ 60 min \| .918 \| .704 \| 1.197 \| .53 \| \| Smartphone usage ≥ 1 h/d \| 1.341 \| 1.012 \| 1.778 \| .041 \| \| Work spent farming \| .996 \| .988 \| 1.004 \| .29 \| \| Work spent outside farming \| 1.005 \| .994 \| 1.016 \| .40 \| \| Farm employment type (part-time vs. full time) \| .740 \| .495 \| 1.107 \| .14 \| \| Farming method \|  \|  \|  \| .39 \| \| Farming method (conventional vs. organic) \| 1.163 \| .820 \| 1.648 \| .40 \| \| Farming method (integrated vs. organic) \| .930 \| .694 \| 1.247 \| .63 \| \| Farm size \|  \|  \|  \| .34 \| \| Farm size (10 to < 20 ha vs. <10 ha) \| .701 \| .399 \| 1.232 \| .22 \| \| Farm size (20 to < 30 ha vs. <10 ha) \| .711 \| .409 \| 1.237 \| .23 \| \| Farm size (30 to < 50 ha vs. <10 ha) \| .982 \| .578 \| 1.667 \| .95 \| \| Farm size (50 to < 100 ha vs. <10 ha) \| .740 \| .421 \| 1.301 \| .30 \| \| Farm size (≥100 ha vs. <10 ha) \| .732 \| .365 \| 1.466 \| .38 \| \| Animal husbandry (yes vs. no) \| .756 \| .467 \| 1.225 \| .26 \| \| Financial situation \|  \|  \|  \| .62 \| \| Financial situation (good vs. very good) \| .824 \| .517 \| 1.312 \| .41 \| \| Financial situation (modest vs. very good) \| .753 \| .472 \| 1.201 \| .23 \| \| Financial situation (poor vs. very good) \| .788 \| .470 \| 1.319 \| .36 \| \| Financial situation (very poor vs. very good) \| .576 \| .282 \| 1.176 \| .13 \|   Note: The regions were classified according to NUTS 1 (Nomenclature of territorial units for statistics) into three major socio-economic regions (Eastern Austria: Burgenland. Lower Austria. Vienna; Southern Austria: Carinthia. Styria; Western Austria: Upper Austria. Salzburg. Tyrol. Vorarlberg).  The binomial logistic regression model was statistically significant. Χ²(26) = 115.061. p < .001. Overall percentage of accuracy in classification was 84.3% and the amount of explained variance. As shown by Nagelkerke’s R². was .096. |
| --- | --- | --- | --- | --- | --- | --- | --- | --- | --- | --- | --- | --- | --- | --- | --- | --- | --- | --- | --- | --- | --- | --- | --- | --- | --- | --- | --- | --- | --- | --- | --- | --- | --- | --- | --- | --- | --- | --- | --- | --- | --- | --- | --- | --- | --- | --- | --- | --- | --- | --- | --- | --- | --- | --- | --- | --- | --- | --- | --- | --- | --- | --- | --- | --- | --- | --- | --- | --- | --- | --- | --- | --- | --- | --- | --- | --- | --- | --- | --- | --- | --- | --- | --- | --- | --- | --- | --- | --- | --- | --- | --- | --- | --- | --- | --- | --- | --- | --- | --- | --- | --- | --- | --- | --- | --- | --- | --- | --- | --- | --- | --- | --- | --- | --- | --- | --- | --- | --- | --- | --- | --- | --- | --- | --- | --- | --- | --- | --- | --- | --- | --- | --- | --- | --- | --- | --- | --- | --- | --- | --- | --- | --- | --- | --- | --- | --- | --- | --- | --- | --- | --- | --- | --- | --- | --- | --- | --- | --- | --- | --- | --- | --- | --- | --- | --- | --- | --- | --- | --- | --- | --- | --- | --- | --- | --- | --- | --- | --- | --- | --- | --- | --- | --- | --- | --- | --- | --- | --- | --- | --- | --- | --- | --- | --- | --- | --- | --- | --- | --- | --- | --- | --- | --- | --- | --- | --- | --- | --- | --- | --- | --- | --- | --- | --- | --- | --- | --- | --- | --- | --- | --- | --- | --- | --- | --- | --- | --- | --- | --- | --- | --- | --- | --- | --- | --- | --- | --- | --- | --- | --- | --- | --- | --- | --- | --- | --- | --- | --- | --- | --- | --- | --- | --- | --- | --- | --- | --- | --- | --- | --- | --- | --- | --- | --- | --- | --- | --- | --- | --- | --- | --- | --- | --- | --- | --- | --- | --- | --- | --- | --- | --- | --- | --- | --- | --- | --- | --- | --- | --- | --- | --- | --- | --- | --- | --- | --- | --- | --- | --- | --- | --- | --- | --- | --- | --- | --- | --- | --- | --- | --- | --- | --- | --- | --- | --- | --- | --- | --- | --- | --- | --- | --- | --- | --- |
